# Delirium in Neurocritical Care: Uncovering Undisclosed Psychotropic Substance and Medication Use and Stress Exposure by Hair Analysis

**DOI:** 10.1007/s12028-024-02052-9

**Published:** 2024-07-16

**Authors:** Stefan Yu Bögli, Crescenzo Capone, Markus R. Baumgartner, Boris B. Quednow, Thomas Kraemer, Emanuela Keller, Tina Maria Binz

**Affiliations:** 1 Neurocritical Care Unit, Institute for Intensive Care Medicine and Department of Neurosurgery, University Hospital Zurich, University Zurich, Frauenklinikstrasse 10, 8091 Zurich, Switzerland; 2 Department of Neurology, Clinical Neuroscience Center, University Hospital Zurich, University Zurich, Frauenklinikstrasse 26, 8091 Zurich, Switzerland; 3 Center for Forensic Hair Analytics, Zurich Institute of Forensic Medicine, University of Zurich, Zurich, Switzerland; 4 Experimental and Clinical Pharmacopsychology, Department of Adult Psychiatry and Psychotherapy, Psychiatric University Hospital Zurich, University of Zurich, Zurich, Switzerland; 5 Department of Forensic Pharmacology and Toxicology, Zurich Institute of Forensic Medicine, University of Zurich, Zurich, Switzerland

**Keywords:** Acute brain injury, Delirium, Hair analysis, Neurocritical care

## Abstract

**Objective:**

In intensive care, delirium is frequent, prolongs the stay, increases health care costs, and worsens patient outcome. Several substances and medications as well as stress can impact the risk of delirium; however, assessment of previous exposure to psychotropic agents and stress by self-reports or third-party information is not always reliable. Hair analysis can be used to objectively assess medication and substance use (including chronic alcohol consumption), and allows for the determination of stress-related long-term changes in steroid hormones and endocannabinoids.

**Methods:**

Consecutive adult patients with acute brain injury admitted to the neurocritical care unit were included. Delirium was diagnosed using the Confusion Assessment Method for the Intensive Care Unit. Liquid chromatography coupled with tandem mass spectrometry was used to investigate psychoactive substances and medications, ethyl glucuronide, steroid hormones, and endocannabinoids in hair samples. Univariable and multivariable analyses were used to reveal any associations with the occurrence of delirium.

**Results:**

Of 50 consecutive patients, 21 (42%) were diagnosed with delirium. Detection of antipsychotics or antidepressants in hair was more frequent in patients with delirium (antidepressants: 43% vs. 14%, *p* = 0.040; antipsychotics: 29% vs. 0%, *p* = 0.021). These patients also displayed higher ethyl glucuronide levels (*p* = 0.049). Anandamide (AEA) concentrations were higher in patients with delirium (*p* = 0.005), whereas oleoylethanolamide (*p* = 0.045) and palmitoylethanolamide (PEA) (*p* = 0.017) concentrations were lower in patients with delirium. Backward stepwise logistic regression analysis revealed antidepressants and AEA/PEA to be independent relevant predictors of delirium.

**Conclusions:**

Hair analysis provides crucial and otherwise unattainable information regarding chronic stress and the use of psychotropic substances and medications. Undisclosed antidepressant/antipsychotic use or intense chronic alcohol consumption is susceptible to treatment (continuation of medication or provision of low-dose benzodiazepines in case of alcohol). Chronic stress can be evaluated using stress markers and endocannabinoids in hair, potentially allowing for personalized delirium risk stratification and preventive measures.

**Supplementary Information:**

The online version contains supplementary material available at 10.1007/s12028-024-02052-9.

## Introduction

Delirium is defined as a fluctuating syndrome of the simultaneous occurrence of disturbed attention (i.e., reduced ability to focus, direct, or sustain attention), awareness (reduced orientation to the environment), and cognition (e.g., memory, perception, executive function) [1]. It is important to note that these symptoms occur within a short period of time and are not explained by a preexisting neurocognitive disorder or a severely reduced level of consciousness [1]. Delirium is a frequent syndrome found in intensive care, occurring in around 30% to 80% of patients depending on various predisposing and precipitating factors [2, 3]. In most patients, predisposing (age, frailty, alcohol/drug abuse, preexisting neurocognitive disorder) and precipitating factors (procedures/surgery, metabolic disturbances, environmental changes) coincide, disrupting the patient’s equilibrium and leading to the development of delirium [3, 4]. Delirium increases mortality and hospital length of stay and impairs long-term cognitive function in survivors [5].

Many patients with acute brain injury (ABI) are admitted to the neurocritical care unit (NCCU) unconscious or even unidentified. Thus, evaluation of medication history and possible use of psychotropic medications or illicit substances is arduous. Although relatives may be able to report on the patients’ major medical conditions, even spouses and sometimes the primary care physicians may not be able to provide information on all the medications taken or possible nonadherence. Substance use or illicit use of medications is common in ABI [6–8] and often remains hidden even to the primary care physician because of underreporting [9–11]. Hidden and therefore untreated withdrawal of substances can lead to delirium and, in severe cases, can be life-threatening [12, 13]. Widely available and applied urine sampling can only inform the physician of medications/substances used in the recent hours up to days depending on the pharmacokinetics and dosage taken [14, 15]. In comparison to the short detection window in urine, the detection window in hair can range from weeks to several months prior to sampling depending on the length of the sample.

Acute stress as by the ABI itself or the subsequent immobility and mechanical ventilation are well known delirium risk factors. Stress leads to an activation of the hypothalamic–pituitary–adrenal (HPA) axis, which ultimately increases excretion of glucocorticoids (cortisone, cortisol) [16]. Sex-specific aspects of this response exist, with an inverse relationship between progesterone in women/testosterone in men and the level of stress-induced cortisol [17]. The endocannabinoid (eCB) system includes various molecules that are involved in the mediation of stress responses after acute or chronic stress [18]. The most promising eCBs when assessing stress-related changes include anandamide (AEA), 2-arachidonoylglycerol (2-AG), palmitoylethanolamide (PEA), and oleoylethanolamide (OEA), all of which can be detected using hair analysis [19]. Particularly the changes in response to chronic stress prior to ictus are of particular interest, as this aspect has been less investigated.

Hair samples, when analyzed for psychoactive compounds (such as antidepressants, antipsychotics, or also illicit substances), steroid hormones, and eCB, might provide otherwise inaccessible information, describing accumulated changes caused by stress as well as potential medication/substance intake in the weeks to months before ictus. We hypothesize that use of psychoactive compounds and stress is associated with a higher incidence of delirium. The aim of this study was to, thus, evaluate the use of hair analysis for the prediction of delirium in an NCCU ABI population.

## Methods

The study was approved by the local ethics committee (Cantonal Ethics Committee Zurich, BASEC-Nr. 2021–00971) and was in accordance with the ethical standards laid down in the 2013 Declaration of Helsinki for research involving human study participants. After admission, the patient or the next of kin (in case of missing capacity) was informed of the study, including the use of hair (removed for medical interventions) for further analysis. Informed consent was received before inclusion from the patient or their legal medical representative.

### Patient Population

All consecutive patients with emergency hospitalization due to ABI (aneurysmal subarachnoid hemorrhage [aSAH], intracerebral hemorrhage [ICH], traumatic brain injury [TBI]) were evaluated for inclusion. The specific inclusion criteria were as follows: (1) signed general consent, (2) age ≥ 18 years, (3) intervention with need for hair removal (e.g., evacuation of hemorrhage, external ventricular drain insertion), and (4) suitable hair for evaluation (i.e., exclusion of patients with no hair or bleached/colored hair, evaluated visually and by asking the patient/next of kin).

Patient-related data were prospectively collected by the clinical team blinded for the results of the hair analysis. Clinical data included the following: age, sex, NCCU length of stay, diagnosis (aSAH, ICH, TBI), initial Glasgow Coma Scale (GCS) score, initial modified Rankin Scale (mRS) score, presence of premorbid psychiatric disorders, and availability (within the first 48 h after admission) of any medication list or available documentation of regularly taken medication. Lastly any documented prior use of antidepressants, antipsychotics, benzodiazepines or other sleep aids, opioids, antiseizure medications, or nonopioid pain killers or nonmedical use of medications and substances, including alcohol, was noted.

The primary outcome was defined as the occurrence of delirium (based on the Confusion Assessment Method for the Intensive Care Unit [CAM-ICU] [2] assessed every 8 h during the intensive care unit [ICU] stay). Patients with a Richmond Agitation Sedation Scale (RASS) [20] score of either − 4 or − 5 during the complete NCCU stay were not included in the analysis considering delirium as the primary outcome. Common mimics were excluded during daily practice by the treating physician by systematically screening for infection or metabolic derangements as well as computed tomography scans (exclusion of secondary brain injury after ABI, including symptomatic vasospasm, delayed cerebral ischemia, and rebleeding), which can both mimic but also precipitate delirium. Secondary outcomes included in-hospital mortality as well as the mRS score at 3 and 12 months (assessed during routine out-patient consultations by the treating physicians).

### Hair Analysis

Hair was routinely removed directly above the skin to allow for sterile conditions before neurosurgery in the operating room or during invasive interventions in the NCCU (insertion of central venous catheters, arterial lines, etc.). Hair was preserved within 48 h after admission to the hospital. Hair analysis was performed blinded to the clinical data. The validated methods used for the simultaneous extraction of drugs, pharmaceuticals, and endogenous steroids in hair has been previously described [21]. Briefly, 3 cm of hair (cut directly above the skin) was preprocessed (washed, dried, chopped) and then processed for analysis by liquid chromatography coupled with tandem mass spectrometry to investigate endogenous steroid hormones [22], psychotropic substances and medications [23], and the alcohol metabolite ethyl glucuronide (EtG) [24]. Depending on the method, 10–20 mg of hair is needed for one analysis. The use of 3 cm of hair proximal to the scalp allows for the evaluation of the compounds deposited within the hair structure within the last 12 weeks. Drugs/pharmaceuticals were dichotomized by intake vs. no intake by the analysis team blinded to the patient’s clinical data/medical history. Furthermore, they were grouped into substance types (antidepressants, antipsychotics, benzodiazepines/sleep aids, opioids, illicit substances, antiseizure medications, nonopioid painkillers). The specific compounds per group can be found in Supplemental Table 1. Alcohol consumption was dichotomized with a cutoff of > 30 pg/mg of EtG to diagnose intense chronic drinking and consequently suspected alcohol use disorder (sAUD) [25]. The following substances and medications were excluded from the analysis because of their high use in emergency and critical care and thus potential contamination: midazolam, morphine, fentanyl, pethidine, and hydromorphone. Exogeneous cannabinoids, such as delta-9-tetrahydroxycannabinol, were not measured.

**Table 1:** Patient characteristics

Characteristic	*N* = 50 (100%)
Age	57 ± 16
Sex (male)	26 (52%)
ICU length of stay, days	11 (5–17)
Diagnosis	
ICH	17 (34%)
aSAH	26 (52%)
TBI	7 (14%)
Initial GCS	9 (5–14)
mRS at admission	
1	3 (6.0%)
2	3 (6.0%)
3	7 (14%)
4	11 (22%)
5	26 (52%)
Premorbid psychiatric disorder	4 (8%)
Delirium	
Delirium	21 (42%)
No delirium	21 (42%)
RASS − 4/ − 5	8 (16%)
In-hospital mortality	13 (26%)
3-month outcome (unfavorable)	29 (58%)
12-month outcome (unfavorable)	21 (42%)

Data are shown as mean ± SD, *n* (%), or median (interquartile range)

aSAH, aneurysmal subarachnoid hemorrhage, GCS, Glasgow Coma Scale, ICH, intracerebral hemorrhage, ICU, intensive care unit, mRS, modified Rankin Scale, RASS, Richmond Agitation Sedation Scale, TBI, traumatic brain injury

### Statistical Analysis

Statistical analysis was performed, and plots were produced using R Studio (R version 4.3.2, https://www.r-project.org/; packages used: tidyverse, gtsummary, ggplot2, ggalluvial). Descriptive statistics are reported as counts and percentages, mean ± standard deviation, or as median and interquartile range (IQR) as appropriate. The data were either dichotomized by the presence of delirium or by outcome, with mRS scores 0–2 being favorable and mRS scores 3–6 being unfavorable. Categorical variables were compared using Pearson’s χ^2^ test or Fisher’s exact test, and continuous/ordinal variables were compared using Student’s *t*-test and the Mann–Whitney *U*-test for parametric and nonparametric data, respectively, where appropriate. Multivariate analysis was performed by first fitting a logistic regression model including all possible clinical predictors (i.e., age, sex, diagnosis, GCS score, mRS score at admission, NCCU length of stay, presence of premorbid psychiatric disorders) and the predictors found in hair analysis (separately). The model was then fed through a backward stepwise elimination process, with a *p* value threshold of 0.05 evaluated using the Akaike information criterion for comparison of the different models. The model parameters are described using odds ratios (ORs) and their 95% confidence intervals (CIs). The backward stepwise elimination process was chosen because it allows for (in light of the relatively small number of patients) simplification of the model, retaining only the key drivers of the outcome. A significance level of *p* < 0.05 (no correction for multiple testing) was set because of the exploratory nature of the study, with a large number of different substances explored.

## Results

### Patient Characteristics

Of a total of 57 consecutive patients with ABI, 50 were included in this study. The demographics and disease characteristics are shown in Table 1. The average age was 57 ± 16 years, and 52% were male. The most common diagnosis was aSAH (52%), followed by ICH (34%) and TBI (14%). The median mRS score at admission was 4 (IQR 3–5). Overall, 42% of patients were diagnosed with delirium during the NCCU stay; 16% did not reach a RASS score of higher than − 4 due to stupor or coma while on sedative and analgesic agents, thus precluding delirium from being diagnosed. After 12 months, 58% reached a favorable outcome and 42% had an unfavorable outcome.

### Medication and Substance Use

A variety of substances were detected via hair analysis. A complete list, including all detected compounds, is presented in Supplemental Table 1. The most frequently detected substances were the nonopioid painkillers paracetamol (88%) and diclofenac (50%), followed by illicit substances (cocaine [20%], methamphetamine [16%], ketamine [14%]), antidepressants (including amitriptyline [14%] and fluoxetine, mirtazapine, and trazodone [6%] each), benzodiazepines (including zolpidem and flunitrazepam [8%] each), opioids (including oxycodone and tramadol [8%] each), and antipsychotics (quetiapine [4%] and levomepromazine, olanzapine, pipamperone, and risperidone [2%] each). In all cases, any medication described in the medication history was also detected by hair analysis, proving the sensitivity of hair analysis. Table 2 shows the number of detected substances, including the alcohol marker, depending on type of ABI. Patients with TBI had higher levels of EtG in comparison to patients with aSAH (*p* = 0.014) and had more commonly used benzodiazepines (*p* = 0.042) in the weeks before ictus. Conversely both patients with TBI and patients with aSAH had higher frequencies of illicit substance consumption before ictus in comparison with patients with ICH (*p* = 0.019 and 0.001, respectively).

**Table 2:** Substance and medication use depending on diagnosis

	Diagnosis			*p* values		
	ICH *n* = 17 (34%)	aSAH *n* = 26 (52%)	TBI *n* = 7 (14%)	ICH vs. aSAH	ICH vs. TBI	aSAH vs. TBI
Alcohol marker EtG (pg/mg), median (IQR)	1 (0–153)	1 (0–9)	40 (11–96)	0.7	0.3	0.014
Suspected alcohol use disorder	4 (24%)	3 (12%)	3 (43%)	0.11	0.7	0.074
Antidepressants	6 (35%)	5 (19%)	2 (29%)	0.3	> 0.9	0.6
Antipsychotics	1 (5.9%)	3 (12%)	2 (29%)	> 0.9	0.2	0.3
Benzodiazepines/sleep aids	4 (24%)	4 (15%)	4 (57%)	0.7	0.2	0.042
Benzodiazepines only	3 (18%)	4 (15%)	4 (57%)	> 0.9	0.13	0.042
Opioids	1 (5.9%)	4 (15%)	3 (43%)	0.6	0.059	0.15
Illicit substances	2 (12%)	12 (46%)	6 (86%)	0.019	0.001	0.10
Antiseizure medication	1 (5.9%)	0 (0%)	1 (14%)	0.4	0.5	0.2
Nonopioid painkillers	15 (88%)	24 (92%)	7 (100%)	> 0.9	> 0.9	> 0.9

aSAH, aneurysmal subarachnoid hemorrhage, EtG, ethyl glucuronide, ICH, intracerebral hemorrhage, IQR, interquartile range, TBI, traumatic brain injury

### Medications and Substances Detected vs. Described

Table 3 shows the number of patients in whom either medication or chronic alcohol use was detected vs. described in the available medication history. All medications and substances except for antiseizure medications were less frequently detected in the medication history than detected in the hair analysis (documented vs. detected: antidepressants, 16% vs. 26%; antipsychotics, 4% vs. 12%; benzodiazepine/sleep aid, 4% vs. 24%; opioids, 2% vs. 16%). Illicit substances were detected in 40% of patients but were never described in the patient history. In seven patients (14%), no medication history was available from the next of kin/general practitioner within the first 48 h after admission. Intense chronic alcohol use was previously described in 12% of patients but was detected in 20%. In one patient, problematic alcohol use was documented without a corresponding increase in the EtG level in the hair analysis.

**Table 3:** Medications and illicit substances

	Documented	Detected
Antidepressants	8 (16%)	13 (26%)
Antipsychotics	2 (4%)	6 (12%)
Opioids	1 (2%)	8 (16%)
Benzodiazepines/sleep aids	2 (4%)	12 (24%)
Illicit substances	0 (0%)	20 (40%)
Antiseizure medication	2 (4%)	2 (4%)
Nonopioid painkillers	2 (4%)	46 (92%)
Suspected alcohol use disorder	6 (12%)	10 (20%)

The number and percentage of patients in whom either medication or intense chronic drinking was detected in the hair analysis vs. documented in the available medication history

### Association Between Medications and Substances Detected and Delirium

**Fig. 1:**
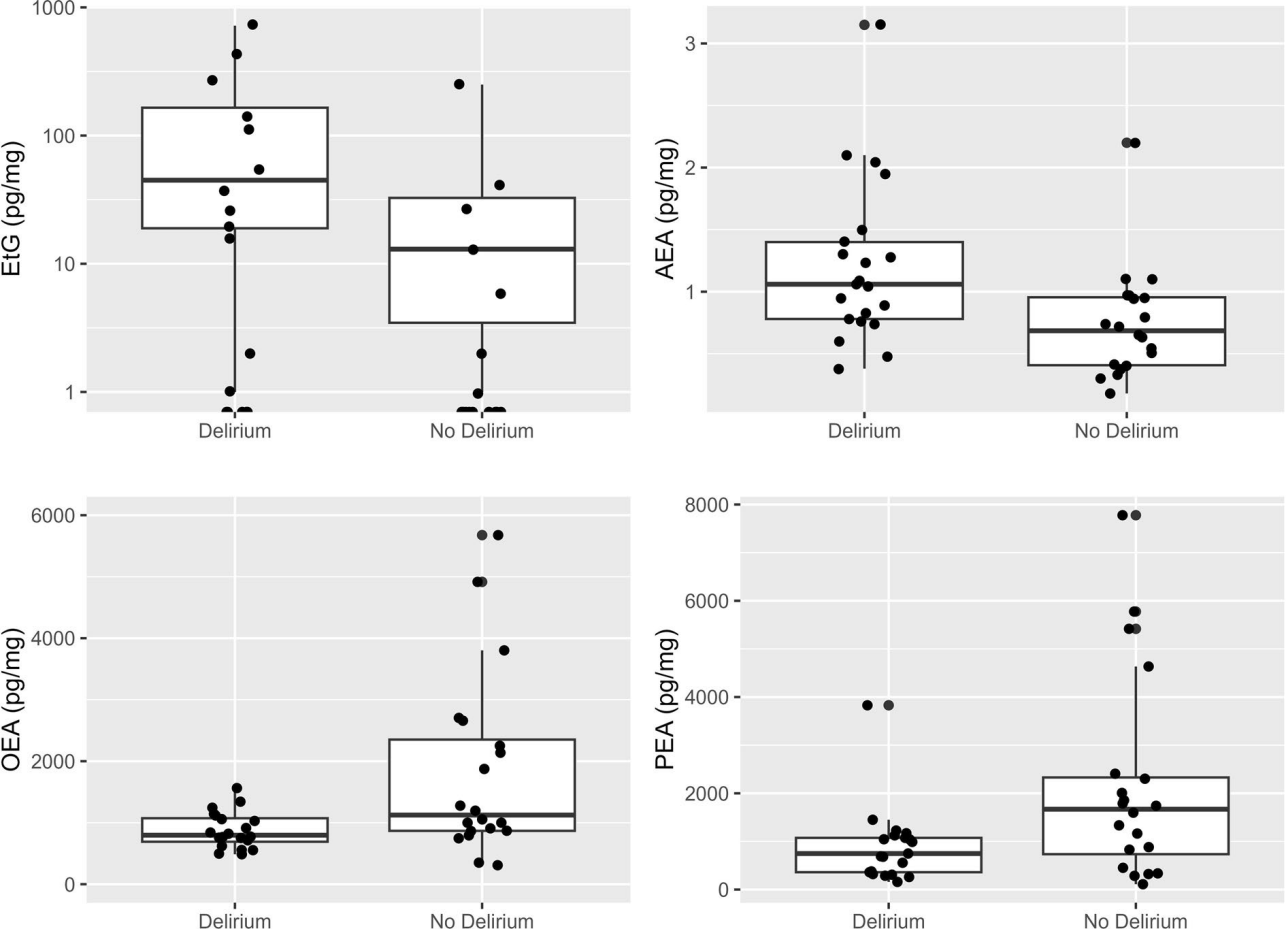
Predictors of delirium. The levels ethyl glucuronide (EtG), anandamide (AEA), palmitoylethanolamide (PEA), and oleoylethanolamide (OEA) by occurrence of delirium

Table 4 shows the associations between the number of detected medications and substances and the incidence of delirium. Overall, antipsychotics and antidepressants were detected more often in patients with delirium (antidepressants: 43% vs. 14% [*p* = 0.040]; antipsychotics: 29% vs. 0% [*p* = 0.021] for patients with and without delirium, respectively). Although sAUD (i.e., EtG level more than 30 pg/mg) was not more frequent in patients with delirium, the absolute amount of EtG was higher in patients with delirium (*p* = 0.049; Fig. 1).

**Table 4:** Substance and medication use association with delirium

	Delirium *n* = 21 (50%)	No delirium *n* = 21 (50%)	*p* value
Alcohol marker EtG (pg/mg), median (IQR)	20 (0–110)	0 (0–8)	0.049
Suspected alcohol use disorder	7 (33%)	2 (9.5%)	0.24
Antidepressants	9 (43%)	3 (14%)	0.040
Antipsychotics	6 (29%)	0 (0%)	0.021
Benzodiazepines/sleep aids	6 (29%)	5 (24%)	0.73
Benzodiazepines only	6 (29%)	4 (19%)	0.47
Opioids	5 (24%)	3 (14%)	0.70
Illicit substances	8 (38%)	9 (43%)	0.75
Antiseizure medication	1 (4.8%)	1 (4.8%)	> 0.99
Nonopioid painkillers	20 (95%)	20 (95%)	> 0.99

EtG, ethyl glucuronide, IQR, interquartile range

### Steroid Hormones and eCBs

**Fig. 2:**
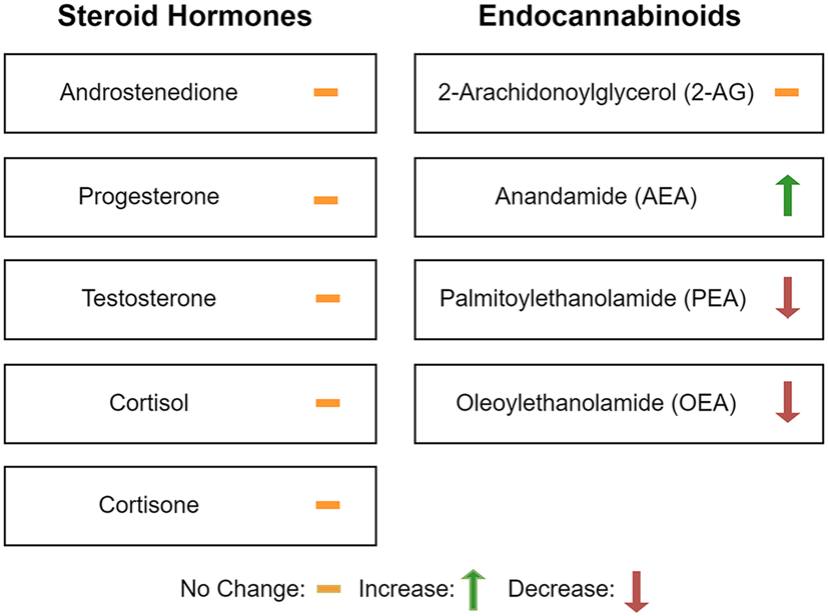
Changes in steroid hormone and endocannabinoid levels depending on the occurrence of delirium

Steroid hormones and eCBs are shown dichotomized by the occurrence of delirium in Table 5. The changes depending on occurrence of delirium are shown in Fig. 2. Overall, there were no differences in the steroid hormones androstenedione, cortisol, cortisone, progesterone, and testosterone when considering the incidence of delirium. However, the eCB AEA level was higher (delirium: median 1.06 pg/mg [IQR 0.78–1.40]; no delirium: median 0.69 pg/mg [IQR 0.41–0.96]; *p* = 0.005) in patients with delirium, and OEA (delirium: median 821 pg/mg [IQR 714–1,118]; no delirium: median 1,124 pg/mg [IQR 868–2,352]; *p* = 0.045) and PEA (delirium: median 748 pg/mg [IQR 360–1,074]; no delirium: median 1,669 pg/mg [IQR 735–2,329]; *p* = 0.017) levels were lower in patients with delirium (Fig. 1).

**Table 5:** Steroid hormones and endocannabinoids associated with delirium

	Delirium *n* = 21 (50%)	No delirium *n* = 21 (50%)	*p* value
Androstenedione (pg/mg), median (IQR)	0.60 (0.35–0.86)	0.61 (0.37–0.90)	0.93
Cortisol (pg/mg), median (IQR)	10 (4–27)	7 (3–18)	0.25
Cortisone (pg/mg), median (IQR)	18 (11–37)	19 (11–33)	0.81
Cortisone and cortisol (pg/mg), median (IQR)	32 (20–61)	27 (15–44)	0.45
Cortisone/cortisol ratio, median (IQR)	2.36 (1.07–3.40)	2.63 (1.77–4.00)	0.28
Progesterone (pg/mg), median (IQR)	0.59 (0.28–0.88)	0.57 (0.31–1.06)	0.96
Testosterone (pg/mg), median (IQR)	0.24 (0.00–0.74)	0.24 (0.00–0.82)	0.92
2-AG (pg/mg), median (IQR)	94 (70–178)	107 (69–154)	0.69
AEA (pg/mg), median (IQR)	1.06 (0.78–1.40)	0.69 (0.41–0.96)	0.005
OEA (pg/mg), median (IQR)	821 (714–1,118)	1,124 (868–2,352)	0.045
PEA (pg/mg), median (IQR)	748 (360–1,074)	1,669 (735–2,329)	0.017

2-AG, 2-arachidonoylglycerol, AEA, anandamide, IQR, interquartile range, OEA, oleoylethanolamide, PEA, palmitoylethanolamide

### Outcome

**Fig. 3:**
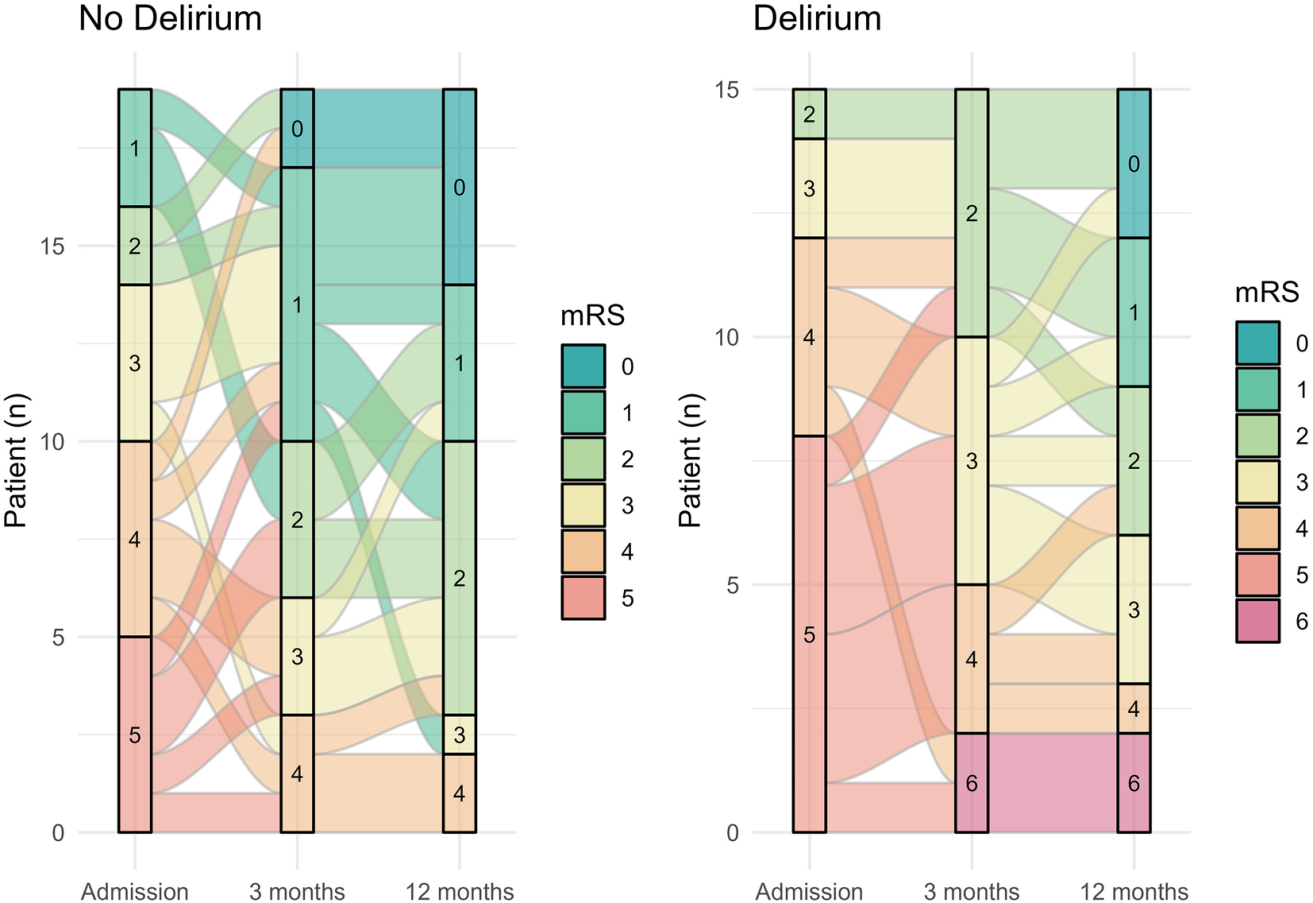
Alluvial plot showing the modified Rankin Scale (mRS) score at admission, 3 months, and 12 months, dichotomized by occurrence of delirium

Overall, patients with delirium fared worse than patients without delirium (Fig. 3). Twenty-nine percent of patients with delirium died during the hospital stay. Unfavorable outcome (i.e., mRS scores 3–6) occurred more commonly in patients with delirium both at 3 months (71% vs. 29%, *p* = 0.014) and at 12 months (48% vs. 14%). None of the medications/eCBs that were found to be predictors of delirium in the prior analysis were direct predictors of unfavorable outcome (Supplemental Tables 2 and 3).

### Multivariable Analysis

The found predictors of delirium were fed individually through a backward stepwise logistic regression analysis (Table 6). The initial model included age, sex, diagnosis, GCS score, mRS score at admission, NCCU length of stay, presence of premorbid psychiatric disorder, and either hair-derived metric. Measured antipsychotics and EtG and OEA levels were dropped during the backward stepwise elimination process. Measured antidepressants (OR 9.47, CI 1.73–77.33) and every 1-pg/mg increase of AEA (OR 7.73, CI 1.61–65.30) were associated with a higher likelihood of developing delirium, whereas every 1,000-pg/mg increase of PEA was associated with a lower likelihood of developing delirium (OR 0.49, CI 0.21–0.86).

**Table 6:** Multivariable backward stepwise logistic regression analysis

	OR (CI)	*p* value
Model including antidepressants		
mRS score at admission (per step increase)	2.22 (1.16–5.24)	0.033
Measured antidepressants (yes)	9.47 (1.73–77.33)	0.018
Model including AEA		
mRS score at admission (per step increase)	2.13 (1.12–4.90)	0.041
AEA (per 1-pg/mg increase)	7.73 (1.61–65.30)	0.025
Model including PEA		
PEA (per 1,000-pg/mg increase)	0.21 (0.04–0.59)	0.019

Initial logistic regression included age, sex, diagnosis, Glasgow Coma Scale score, mRS score at admission, neurocritical care unit length of stay, premorbid psychiatric disorder, and either hair-derived metric (measured antidepressant, AEA, PEA)

AEA, anandamide, CI, confidence interval, mRS, modified Rankin Scale, OR, odds ratio, PEA, palmitoylethanolamide

## Discussion

Hair analysis is feasible in an intensive care setting and may be a useful tool for predicting delirium and for detecting undisclosed use of substances and medications in an NCCU ABI population. This study provides a detailed description of (1) the prevalence of and association between detected prior use of medications and illicit substances and chronic alcohol use and the occurrence of delirium and (2) the associations between steroid hormones and eCBs and delirium.

The number of illicit substances and pharmaceuticals detected vastly exceeded the number expected as well as reported within the medication history. After ICU admission, both the patients’ relatives and their general practitioners/family physicians were contacted for a reliable documentation of prescribed medications. However, increasing availability of medical subspecialties and the possibility of independently choosing the physician in Switzerland are related to the known issue of polypharmacotherapy in the context of multiple prescribers [26, 27]. Antidepressants are part of the commonly prescribed medications in Switzerland, with an average use of 8.7% rising to more than 16% when considering individuals aged 65 or older [28]. Similar results have been described for benzodiazepines and other sleep aids. On average, close to 9% of the Swiss population receives at least one prescription per year (more than 20% of the population aged 65 and above) [29]. Thirty-six percent of the Swiss population aged older than 65 has been prescribed an antipsychotic medication [30]. Overall, the frequencies of pharmaceuticals found reflect the prescription rates, yet the underreporting is concerning and further underlines the importance of prescriber–prescriber dialogue [26].

Incomplete medication history can lead to inadvertent withdrawal. Of particular concern are patients with undisclosed use of benzodiazepines or opioids. In addition to increasing mortality, chronic use of opioids and other anesthesia drugs is associated with various complications in critical care, including tolerance to anesthetics [31, 32]. The acute withdrawal of benzodiazepines can lead to derangement of the sleep–wake cycle and delirium. Accordingly, nonmedical benzodiazepine use is associated with the need for higher sedative dosages in critical care to attain sufficient sedation depth [33]. In our cohort, the detection of the use of antidepressants or antipsychotics was associated with an increased incidence of delirium. Depression itself is a known predictor of delirium [34]. Given that 39% of antidepressant use and 67% antipsychotic use were undocumented, many patients received an inadvertent discontinuation during the NCCU stay. The effects of abrupt cessation of antidepressant therapy on patients in intensive care have been well described [35]. Antipsychotics are frequently used for the treatment of delirium in intensive care [36], but withdrawal of them can also trigger delirium [37].

The number of patients with illicit substances (40%) and with sAUD (20%) found in our study was high. Unsurprisingly, and consistent with prior reports [6, 38], patients with TBI were most commonly found to be involved in illicit substance or chronic alcohol use. In patients with aSAH, use of illicit substances (i.e., cocaine, methamphetamine) is less frequent (described in around 5–10%) but is nonetheless an important predictor of unfavorable outcome and higher frequency of complications (i.e., vasospasm) [39–41]. The undisclosed use of illicit substances and problematic alcohol use have a variety of possible adverse effects. In patients with known alcohol use disorder, benzodiazepine-based treatment can be used for the prevention of withdrawal symptoms and delirium [42]. Some of the substances detected are closely related to an inferior outcome or even the occurrence of specific treatable complications, such as vasospasm in aSAH [39] and pneumonia in TBI [43], thus potentially warranting a closer monitoring of these patients. Patients with atypical hemorrhages, even after exclusion of trauma, ruptured aneurysm, or arteriovenous malformation, may undergo additional various diagnostic procedures that might be unnecessary when substance abuse is found [44, 45]. The possible implications for clinical management in case undisclosed use of substances is detected extend beyond the types of ABI covered by this study. In ischemic stroke, for example, cocaine is associated with a unique stroke mechanism, including vasospasm and hypertensive episodes, and an overall worse outcome [46].

Acute stress or stressors (due to the initial ictus itself or precipitating factors such as undertreated pain, oversedation, mechanical ventilation, immobility, or unknown surrounding) are well known triggers of delirium [47]. Prevention methods are well described and widely applied [48]. However, the role of chronic stress prior to incident is less well studied. Glucocorticoids are neuroendocrine transmitters released via stress-related activation of the HPA axis that activate mobilization of energy and suppress nonvital functions. Increased plasma glucocorticoids after an acute stressor, such as surgery, predicts delirium [49]; furthermore, an impaired regulation of the HPA system leads to a decreased resistance of delirium [50]. In our cohort, no chronic elevation of corticosteroids in hair could be found, thus indicating that the patients with delirium might not have suffered from more acute stress than patients without delirium within the weeks before the incident.

eCBs are integral for the regulation of the HPA axis in response to stress. We found that patients with delirium after ABI had higher AEA levels but lower OEA and PEA levels, whereas 2-AG was unchanged. In humans, acute stress exposure leads to increased levels of AEA, PEA, and OEA [51]. Higher levels of PEA and OEA are positively correlated with perceived stress [52]. In patients who are subject to chronic stress, such as due to posttraumatic stress disorder, the stress severity is negatively correlated with the levels of PEA and OEA [53]. Low PEA levels, in addition to reflecting chronic stress, have also been linked to lower stress resilience [54]. Of note, eCB alterations in patients with delirium are likely not explained by the use of cocaine, which was highly prevalent in the present sample, given that cocaine use is associated with changes in 2-AG but not in AEA, PEA, and OEA [55]. Considering these reports, our results suggest that patients with prior chronic stress were more likely to suffer from subsequent delirium after ABI.

## Limitations

This is a single-center study with a moderate sample size. Patients included had a severe ABI warranting an intervention; thus, the results may be limited in terms of generalizability. Considering the exploratory nature of this study and the sensitive topic of scalp hair removal, only patients in whom hair removal was necessary (and who had scalp hair) for an intervention were evaluated for inclusion, resulting in a selection bias. The need for scalp hair can be circumvented by using body hair, as suggested by the Society of Hair Testing [56]. In patients with short hair, a larger section can be sampled. If hair removal is unnecessary in terms of neurosurgery or ICU treatment, a thin hair strand is sufficient for analysis and could be easily taken without leaving obvious cosmetical damage (hair removal). In general, hair growth rates of 1 cm per month are accepted. Individual deviations exist, with growth rates varying between 0.7 to 1.4 cm per month. Bleaching and dyeing (confirmed visually and by asking the patient/next of kin) reduces the detectability of many substances in hair [57]; thus, patients with bleached or dyed hair were excluded whenever possible. Various additional factors can affect the hair analysis, including damaging of the hair due to heat, light, or cosmetics, which cannot be mitigated. Furthermore, although acquiring the results from hair analysis is possible within 48 h, it is currently not part of the routinely offered laboratory analysis provided by the Center for Forensic Hair Analytics. However, because the information is otherwise often unattainable and because delirium risk increases with time spent within an NCCU environment, delayed information might still be relevant for the treatment regimen.

Contamination leads to passive exposure of the hair. Its effect was mitigated by appropriate decontamination procedures in the preprocessing [23, 58]. Wherever possible, the intake of specific substances was validated by the detection of the metabolites (see Supplemental Table 1) and calculation of the metabolite ratios. For some substances, this can be helpful to distinguish between external contamination and intake of a substance (e.g., cocaine). Substances were reported as positive when they measured above the method-specific limit of quantification. Associations with substances/pharmaceuticals were made by dichotomization, thus not taking the absolute level into account. A dose-dependent and substance-specific effect is likely but could not be further elucidated because of the sample size. Of note, we decided to classify ketamine as an illicit substance. However, although unlikely (considering the medication history and the available patient reports) patients could have been exposed to ketamine as a medical treatment (e.g., for major depression or chronic pain). Lastly, delirium is a complex disease with various predisposing and precipitating factors that exceed the ones presented in this study but might alter the results found.

### Future Directions

The current study was planned as an observational study because of lack of studies describing feasibility and utility of hair analysis in this patient cohort. Consequently, the results primarily describe associations. Both teams (clinical vs. hair analysis) acquired the data sets independently and were blinded to the results generated by the other team. Our results show compelling evidence that withdrawal of medications or substances might have escalated the delirium risk. Future studies should aim to expand the cohort of patients included (e.g., by circumventing the sensitive topic of using scalp hair via the use of body hair, as suggested by the Society of Hair Testing) and evaluate whether an intervention (i.e., restarting the medication or, in case of alcohol withdrawal, use of preventive measures) mitigates the risk of delirium. For clinical applications, it would be desirable to develop quick hair extraction protocols that allow for rapid analysis within 24 h. Using current knowledge and state-of-the-art analytical instruments, this should be possible and would pave the way to use hair analysis in clinical settings.

## Conclusions

Hair analysis provides crucial and otherwise unattainable information regarding chronic stress and the use of substances and medications. Detected (and often not reported/inadvertently discontinued) use of antidepressants and antipsychotics and problematic alcohol use predicts occurrence of delirium and is susceptible to treatment (continuation of medication or provision of low-dose benzodiazepines in case of chronic excessive alcohol consumption). Chronic stress evaluated using eCBs predicts delirium, thus, potentially allowing for personalized risk stratification. The potential benefits of hair analysis extend beyond the immediate implications for the prediction and mitigation of delirium considering the importance of the various substances found for the treatment of various types of brain injury.

## Supplementary Information

Below is the link to the electronic supplementary material.

Supplementary file1 (DOCX 49 kb)

## Data Availability

The postprocessed data are available upon reasonable request to the corresponding author.
