# Supplementary material for: Delirium in Neurocritical Care: Uncovering Undisclosed Psychotropic Substance and Medication Use and Stress Exposure by Hair Analysis
Source: Neurocrit Care. 2024 Jul 16;42(1):164–74. doi: 10.1007/s12028-024-02052-9 (PMC11811262; doi:10.1007/s12028-024-02052-9)
Supplement: Supplementary file 1 — Supplementary file1 (DOCX 49 kb) [file 12028_2024_2052_MOESM1_ESM.docx]

# **SUPPLEMENT**

**Supplemental Table 1. Specific substances and metabolites found in the hair analysis.***

| **Antidepressants** | **n (%)** | **Bezodiazepines/Sleep-aids** | **n (%)** | **Opioids** | **n (%)** |
| --- | --- | --- | --- | --- | --- |
| *Amitryptiline* | 7 (14) | *Clobazam* | 1 (2) | *Codeine* | 2 (4) |
| *Nortryptiline* | 2 (4) | *N-Desalkylclobazepam*† | 1 (2) | *Hydrocodone* | 2 (4) |
| *Bupropion* | 1 (2) | *Diazepam* | 1 (2) | *Oxycodone* | 4 (8) |
| *Citalopram* | 2 (4) | *Nordazepam* | 1 (2) | *Noroxycodone*† | 2 (4) |
| *Fluoxetine* | 3 (6) | *Oxazepam* | 1 (2) | *Oxymorphone* | 2 (4) |
| *Mirtazapine* | 3 (6) | *Temazepam* | 1 (2) | *Tapentadol* | 1 (2) |
| *Sertraline* | 1 (2) | *7-Amino-Flunitrazepam*† | 4 (8) | *Tramadol* | 4 (8) |
| *Trazodon* | 3 (6) | *Lorazepam* | 2 (4) | *N-Desmethyltramadol*† | 4 (8) |
| *Trimipramine* | 1 (2) | *Tetrazepam* | 1 (2) | *Dextromethorphane* | 1 (2) |
| *Venlafaxine* | 1 (2) | *Zolpidem* | 4 (8) |  |  |
| *O-Desmethylvenlafaxine*† | 1 (2) | *Diphenhydramin* | 2 (4) | ***Antiseizure medications*** | **n (%)** |
|  |  |  |  | *Lamotrigine* | 2 (4) |
| **Antipsychotics** | **n (%)** | **Illicit Substances** | **n (%)** |  |  |
| *Levomepromazine* | 1 (2) | *Cocaine* | 10 (20) | ***Non-Opioid Painkillers*** | **n (%)** |
| *Olanzapine* | 1 (2) | *Benzoylegconine*† | 9 (18) | *Diclofenac* | 25 (50) |
| *Pipamperone* | 1 (2) | *Norcocaine*† | 10 (20) | *Paracetamol* | 44 (88) |
| *Quetiapine* | 2 (4) | *Ethylcocaine*† | 9 (18) |  |  |
| *Norquetiapine*† | 1 (2) | *Amphetamine* | 3 (6) | **Other** | **n (%)** |
| *Risperidone* | 1 (2) | *Methamphetamine* | 8 (16) | *Levamisol* | 4 (8) |
| *OH_Risperidone*† | 2 (4) | *MDMA* | 2 (4) |  |  |
|  |  | *MDA* | 2 (4) |  |  |
|  |  | *Ketamine* | 7 (14) |  |  |
|  |  | *Norketamine*† | 7 (14) |  |  |
|  |  |  |  |  |  |

*The following substances were not found but were part of the liquid chromatography coupled to tandem mass spectrometry panel protocol: Anti-depressants: Agomelatine, Clomethiazole, Clomipramine, Doxepin, Duloxetine, Fluoxetine, Fluvoxamine, Imipramine, Opipramole, Paroxetine, Vortioxetine; Neuroleptics: Amisulpride, Aripiprazole, Asenapine, Chlorprothixen, Clozapin, Haloperidon, Promazine; Sleepaids: Alprazolam, OH-Alprazolam, Bromazepam, OH-Bromazepam, Clonazepam, 7-Aminoclonazepam, Demoxepam, Flunitrazepam, N-Desalkylflurazepam, Lormetazepam, Nitrazepam, 7-Amino-Nitrazepam, Phenazepam, Prazepam, Triazolam, Zaleplon, Zopiclone, Doxylamine, Hydroxyzine; Opioids: Monoactelymorphine, Hydromorphone, Acetylcodeine, Dihydrocodeine, Naloxone, Tilidine, Methadone, EDDP, Buprenorphine, Norbuprenorphine; Illicit Substances: MDEA, Methylphenidate, Modafinil; Anti-Seizure Medication: Pregabalin.

†These substances are metabolites

**Supplemental Table 2. Substances and Alcohol Abuse association to 12-month outcome. ***

| **Characteristic** | **Favorable**  N=29 (58%) | **Unfavorable**  N=21 (42%) | **p-value** |
| --- | --- | --- | --- |
| **Alcohol marker EtG (pg/mg)** | 4 (0, 37) | 1 (0, 11) | 0.82 |
| **Suspected alcohol use disorder** | 6 (21%) | 4 (19%) | 0.40 |
| **Antidepressants** | 6 (21%) | 7 (33%) | 0.31 |
| **Antipsychotics** | 3 (10%) | 3 (14%) | 0.69 |
| **Benzodiazepines/sleep-aid** | 9 (31%) | 3 (14%) | 0.17 |
| **Benzodiazepines only** | 8 (28%) | 3 (14%) | 0.32 |
| **Opioids** | 5 (17%) | 3 (14%) | >0.99 |
| **Illicit Substances** | 15 (52%) | 5 (24%) | 0.05 |
| **Antiseizure medications** | 1 (3.4%) | 1 (4.8%) | >0.99 |
| **Non-opioid painkillers** | 28 (97%) | 18 (86%) | 0.30 |

*Abbreviations: ethylglucuronide (EtG)

**Supplemental Table 3. Steroid hormones and endocannabinoids depending on 12-month outcome. ***

| **Characteristic** | **Favorable**  N=29 (58%) | **Unfavorable**  N=21 (42%) | **p-value** |
| --- | --- | --- | --- |
| **Androstenedione (pg/mg)** | 0.60 (0.35, 0.74) | 0.79 (0.36, 1.44) | 0.31 |
| **Cortisol (pg/mg)** | 9 (3, 20) | 10 (5, 31) | 0.29 |
| **Cortisone (pg/mg)** | 21 (10, 32) | 19 (15, 45) | 0.35 |
| **Cortisone and Cortisol (pg/mg)** | 32 (14, 47) | 33 (20, 66) | 0.29 |
| **Cortisone/cortisol ratio** | 2.20 (1.42, 3.98) | 2.65 (1.20, 3.77) | 0.93 |
| **Progesterone (pg/mg)** | 0.62 (0.29, 1.01) | 0.43 (0.24, 0.82) | 0.33 |
| **Testosterone (pg/mg)** | 0.29 (0.00, 0.77) | 0.22 (0.00, 1.34) | 0.58 |
| **2-AG (pg/mg)** | 101 (69, 165) | 94 (83, 122) | 0.96 |
| **AEA (pg/mg)** | 0.83 (0.54, 1.06) | 0.88 (0.57, 1.61) | 0.36 |
| **OEA (pg/mg)** | 1,000 (821, 2,138) | 1,016 (749, 1,126) | 0.35 |
| **PEA (pg/mg)** | 1,074 (557, 2,007) | 719 (303, 1,240) | 0.065 |

*Abbreviations: anandamide (AEA), 2-arachidonoylglycerol (2-AG), palmitoylethanolamide (PEA), and oleoylethanolamide (OEA).
